# An Extension of RE-AIM to Enhance Sustainability: Addressing Dynamic Context and Promoting Health Equity Over Time

**DOI:** 10.3389/fpubh.2020.00134

**Published:** 2020-05-12

**Authors:** Rachel C. Shelton, David A. Chambers, Russell E. Glasgow

**Affiliations:** ^1^Department of Sociomedical Sciences, Mailman School of Public Health, Columbia University, New York, NY, United States; ^2^Division of Cancer Control and Population Sciences, National Cancer Institute, National Institutes of Health, Bethesda, MD, United States; ^3^School of Medicine, University of Colorado Denver, Aurora, CO, United States; ^4^VA Eastern Colorado Health Care System, Aurora, CO, United States

**Keywords:** RE-AIM, sustainability, sustainment, frameworks, health equity, implementation science, evaluation, adaptation

## Abstract

RE-AIM is a widely adopted, robust implementation science (IS) framework used to inform intervention and implementation design, planning, and evaluation, as well as to address short-term maintenance. In recent years, there has been growing focus on the longer-term sustainability of evidence-based programs, policies and practices (EBIs). In particular, investigators have conceptualized sustainability as the continued health impact and delivery of EBIs over a longer period of time (e.g., years after initial implementation) and incorporated the complex and evolving nature of context. We propose a reconsideration of RE-AIM to integrate recent conceptualizations of sustainability with a focus on addressing dynamic context and promoting health equity. In this Perspective, we present an extension of the RE-AIM framework to guide planning, measurement/evaluation, and adaptations focused on enhancing sustainability. We recommend consideration of: (1) extension of “maintenance” within RE-AIM to include recent conceptualizations of dynamic, longer-term intervention sustainability and “evolvability” across the life cycle of EBIs, including adaptation and potential de-implementation in light of changing and evolving evidence, contexts, and population needs; (2) iterative application of RE-AIM assessments to guide adaptations and enhance long-term sustainability; (3) explicit consideration of equity and cost as fundamental, driving forces that need to be addressed across RE-AIM dimensions to enhance sustainability; and (4) use or integration of RE-AIM with other existing frameworks that address key contextual factors and examine multi-level determinants of sustainability. Finally, we provide testable hypotheses and detailed research questions to inform future research in these areas.

## Introduction

RE-AIM is a robust framework that has been widely applied over the past 20 years across a range of public health, clinical, community, and behavioral settings (1–4). RE-AIM was created to help address the well-documented research-to-practice gap that hinders the reduction of health inequities and widespread population health impact. It is one of the most commonly applied frameworks in public health, health behavior, and implementation science (IS) (2–5). RE-AIM can facilitate transparent reporting (1) and enhance planning for successful dissemination and implementation of evidence-based interventions, programs, practices, and policies (“EBIs”). Recent years have seen expansion of RE-AIM to address contextual factors (e.g., RE-AIM/PRISM); (4, 6) and integrate qualitative methods (7, 8).

As a framework, RE-AIM has both individual-level and staff/setting-level dimensions, including Reach and Effectiveness (individual-level), Adoption and Implementation (staff and setting levels), and Maintenance (both individual and staff/setting levels). Recognizing sustained delivery and impact of EBIs as central challenges across settings, RE-AIM has historically been one of the few IS frameworks that explicitly built in measurement and consideration of “maintenance.” “Maintenance” in RE-AIM has been operationalized at the individual level (e.g., long-term effectiveness or impact of EBI) and the setting level (e.g., sustainability of EBI program components after original implementation). The “maintenance” dimension of RE-AIM has typically been assessed at relatively short-term intervals (e.g., 6 months after EBI delivered or initially implemented) and its evaluation has focused on the extent to which a program/policy becomes *institutionalized* (e.g., made part of routine organizational practices and policies) (4).

Within IS, there is growing recognition of the importance of understanding and addressing longer-term sustainability of EBIs (9–12). Achieving sustained impact and delivery of EBIs over time has been identified as one of the most important yet understudied challenges across settings, populations, and health issues (9, 10, 13, 14). There is growing consensus on conceptualizations and definitions of sustainability; e.g., Moore et al. (15) described sustainability as “after a defined period of time, the program, clinical intervention, and/or implementation strategies continue to be delivered and/or individual behavior change (i.e., clinician, patient) is maintained; the program and individual behavior change may evolve or adapt while continuing to produce benefits for individuals/systems.” Of note, we recognize that the terms sustainment and sustainability are both used in reference to the outcome of an intervention being delivered over time, as well as the characteristics of the intervention that make it more likely to be delivered over time. For this paper, we used the term “sustainability” to refer to both the desired outcome and the characteristics or processes by which it is more likely maintained.

There has been an important shift away from “static” conceptualizations of sustainability, with awareness that this may impede adoption of more effective practices as the environment changes or new evidence emerges. Investigators also increasingly recognize the need for a dynamic conceptualization of sustainability, in light of complex “real-world” contexts in which EBIs are delivered that require responsiveness, capacity building, and adaptation of EBIs (10, 11, 16). This is consistent with the Dynamic Sustainability Framework (DSF) (17), which focuses on continued learning and evaluation, problem-solving, improvement and ongoing adaptation of EBIs to enhance fit with contexts and populations. Just as a balance between fidelity and adaptation is needed to achieve “fit” in the context of pre-implementation and implementation efforts (18, 19), there is a similar balance between sustainability of original EBIs and ongoing “evolvability” to achieve ongoing fit and sustained population health impact within broader communities or health systems. Evolvability (20) relates to the adaptation of EBIs and implementation strategies in response to changing contexts and resources over time, as well as emerging needs and evidence across the life cycle of an EBI. This includes both the systematic, planned adaptation of EBIs and strategies, as well as ongoing refinement of EBIs and strategies organically within specific community or clinical settings. Over an EBI's life cycle, this evolution within a changing system or organization may ultimately involve “de-implementation,” or the removal or replacement of EBIs that no longer fit or are ineffective (21, 22).

As explicated below, equity and costs are foundational driving forces across RE-AIM dimensions that shape sustained impact, and warrant the need for initial and ongoing adaptation. EBIs can only succeed at the population health level if they are affordable across most settings and are delivered routinely and equitably over time across diverse settings and populations. As we consider the life cycle of an intervention (18), it may be less useful to think about “sustainability” of the original EBIs as an “end goal” (17), and instead consider “evolvability” across the dynamic life cycle of the EBI within a broader context or system, with the goal of sustainable and equitable health impact.

Important gaps persist in existing frameworks' ability to provide guidance in concretely conceptualizing, measuring/operationalizing, and planning for longer-term sustainability within a dynamic context. For example, RE-AIM does not capture such dynamic conceptualizations of sustainability, and has often been applied as a “one-time” evaluation and planning tool. Given the numerous conceptual frameworks and models in IS (2, 23, 24), we did not seek to create a new framework. Instead, we propose an expansion of RE-AIM to enhance sustainability by focusing on key issues across RE-AIM dimensions, with the goal of increasing health impact and health equity over time.

The purposes of this article are to: (1) discuss the extension of RE-AIM to address dynamic conceptualization of sustainability over time, including iterative application of RE-AIM to guide adaptation and evolvability of EBIs and implementation strategies; (2) provide concrete guidance on issues pertinent to understanding, measuring, and planning for sustainability in changing context, including explicit consideration of costs and equity; and (3) propose testable hypotheses and detailed research questions to guide future research that applies RE-AIM for sustainability.

### Applying Re-Aim to Enhance Sustainability

**Figure 1:**
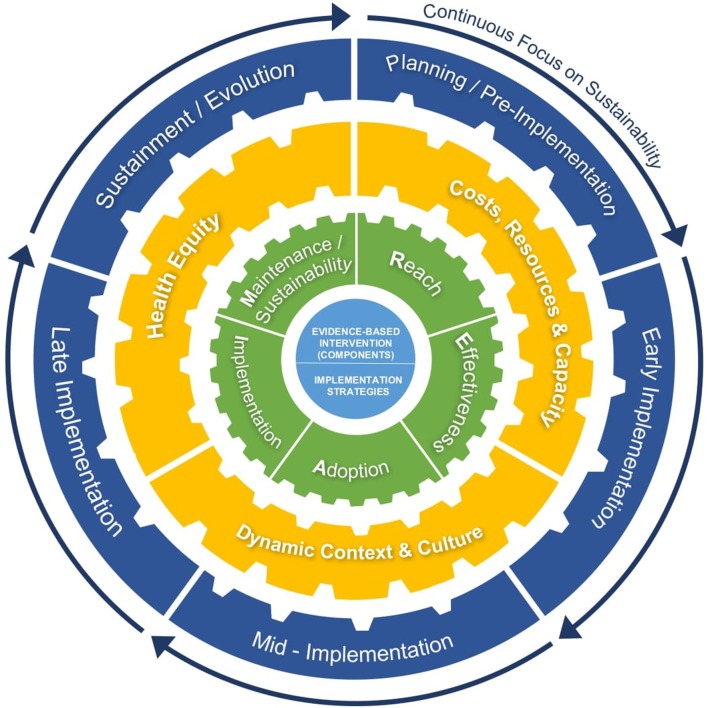
An extension of RE-AIM to enhance sustainability: Cross-cutting issues and iterative application of RE-AIM for sustainability, to guide adaptations and evolvability of EBIs/implementation strategies, address dynamic context, and promote equity across the life cycle of an EBI.

The following sections discuss and provide recommendations to guide planning, adaptation, and measurement when applying RE-AIM to facilitate sustainability, reflecting dynamic sustainability with a focus on context and equity. Each section concludes with example hypotheses to guide research. Five key issues are discussed below, and summarized in Table 1 and Figure 1.

**Table 1:** Iterative application and operationalization of RE-AIM for Sustainability, with a focus on health equity and dynamic context over time.

**Reach**
*Indicators:* Number, proportion, representativeness of individuals who participate in EBI. *Key Questions:* Who was the intended audience and who actually participated? Why or why not? How can we better reach them and engage with them? *Health Equity Considerations:* Are all populations equitably reached by the EBI? Who is not reached by the EBI (in terms of a range of social dimensions and social determinants of health) and why? How can we better reach those who are not receiving the EBI and ensure we are reaching those who experience inequities related to social dimensions and social/structural determinants of health? *Sustainability Considerations:* Who is/isn't reached by the EBI at various time points over time? (e.g., iterative measurement of Reach). Why or why not?
**Effectiveness**
*Indicators:* The impact of an intervention on important health behaviors or outcomes, including quality of life (QOL) and unintended negative consequences; consider heterogeneity of effects. *Key Questions:* Is the EBI effective? For whom? Are there any negative and/or unintended effects? *Health Equity Considerations:* Are the health impacts experienced equitable across all groups on the basis of various social dimensions and social/structural determinants of health- why or why not? Do certain groups experience higher levels of negative effects or burdens? *Sustainability Considerations:* Does the EBI continue to be effective at various time points over time? Among whom?
**Adoption**
*Indicators:* The number, proportion, and representativeness of: (a) settings; and (b) staff/interventionists who deliver the program, including reasons for adoption or non-adoption across settings and interventionists. *Key Questions:* Where was the EBI applied and by who? Which sites/staff were invited and which excluded? Which participated and not? Why? How can the setting/context/staff be better supported to deliver the EBI? *Health Equity Considerations:* Did all settings equitably adopt the EBI? Which settings and staff adopted and applied the EBI? Which did not and why? Were low-resource settings able to adopt the EBI to the same extent as higher-resource settings? What adaptations might be needed to facilitate adoption? *Sustainability Considerations:* Which settings/staff continue to deliver the EBI over time? Which do not and why?
**Implementation**
*Indicators:* At multiple setting and staff levels, continued and consistent delivery of the EBI (and implementation strategies) as intended (fidelity), as well as adaptions made and costs of implementation. *Key Questions:* Was the EBI and/or implementation strategies delivered consistently- why or why not? How was it be adapted and why? How much did it cost? How can we ensure the key functions of the EBI are delivered? Informed by existing implementation frameworks (e.g., PRISM, CFIR), what multi-level contextual determinants matter for implementation? *Health Equity Considerations:* Were the EBI and implementation strategies equitably delivered across settings/staff? Which settings/staff successfully delivered the EBI and implementation strategies and which did not and why? Do all settings/staff have the capacity and resources to deliver the EBI on an ongoing basis? What adaptations might be needed to promote equity and address social determinants of health? *Sustainability Considerations:* How do we ensure that the EBI continues to be delivered consistently over time, especially in the context of reduced funding? Are certain implementation strategies more likely to sustain EBIs and have sustained impact than others?
**Maintenance/Sustainability**
*Indicators:* Extent to which (*a*) health impact/benefits, outcomes, behaviors continue for patients/consumers at the individual level, including patterns in health inequities over time; (*b*) program activities or core components/functions of the original EBI (and strategies) continue to be delivered at setting/staff level with fidelity (e.g., continuation of active ingredients and essential functions/related activities), as well as the “evolvability” of the EBI and implementation strategies needed to support EBI delivery over time, including adaptations (planned and organic) and why they occur; (*c*) community and organizational capacity and infrastructure to deliver the EBI are maintained, including partnerships, networks, and coalitions; and when applicable (d) institutionalization, or extent to which EBI becomes part of routine organizational practices/policies (when considered dynamically over time) (all above measured initially 6 months after initial implementation and at least 1 year post EBI implementation and on ongoing basis, e.g., quarterly to annually). For the above, includes proportion and representativeness of settings that continue EBI and reasons why/not. *Key Questions:* What sustainability strategies can be used to sustain the program long-term beyond 1 year after implementation and longer? What are the costs and return on value of sustainability of an EBI? How can we support and incorporate the EBI so it is delivered past initial implementation or after the funding is over? Informed by existing sustainability frameworks (e.g., PSAT, ISF), what multi-level contextual determinants matter for sustainability? *Health Equity Considerations:* Is the EBI being equitably sustained? What settings and populations continue to be reached long-term by the EBI and continue to receive benefits over time- why or why not? Do adaptations to EBIs reduce or exacerbate health inequities over time? Do all settings have continued capacity and partnerships to maintain delivery of EBIs? Are the determinants of sustainability the same across low-resource and high-resource settings? How do social determinants of health shape inequitable implementation and sustainability of EBIs over time? *Sustainability Considerations:* As the program continues and the context and evidence changes, what adaptations (to the program, strategies, and setting) are needed to continue delivering the EBI long-term? Are there opportunities to build capacity at sites with low maintenance to promote longer-term sustainability? What would it take for sites to sustain the EBI over the long term? What are key multi-level barriers to continued program sustainability over time among a range of stakeholders? What are factors or strategies that might support continuation of the program? Over time as evidence changes, is de-implementation of some program elements a more appropriate outcome than continued delivery of the program? Are there sustainability strategies that are effective at maintaining impact and delivery over time?

#### 1. Extending and Reframing “Maintenance” Within RE-AIM to Include Recent Conceptualizations of Sustainability as an Outcome

Given growing consensus of sustainability as dynamic in nature, it is important that indicators of sustainability reflect this longer-term conceptualization. While 6 months, as originally proposed in RE-AIM (1), is useful in providing an indicator of early maintenance, a more comprehensive approach to also capturing sustainability over time includes measurement at least 1 year post initial implementation and over time (e.g., quarterly to annually) (9, 10).

Consistent with recent conceptualizations (10–12), we recommend that operational indicators of maintenance/sustainability include (see Table 1 for details): (1) extent to which the core components/functions of EBIs and implementation strategies continue to be delivered over time with fidelity (e.g., continuation of active ingredients and essential functions/related activities) (25, 26), and the “evolvability” of the EBI and implementation strategies (27) needed to support continued EBI delivery over time, including adaptations (planned and organic) and why they occur; (2) extent to which the EBI has continued impact on health behaviors/outcomes, when feasible, including patterns in health inequities over time (e.g., who continues to experience health benefits and who does not); and (3) extent to which community and organizational capacity and infrastructure to deliver the EBI are maintained, including partnerships, networks, and coalitions. It is critical to actively engage with stakeholders (e.g., community members, implementers, organizational leaders) to prioritize which maintenance/sustainability outcomes will be measured and when (e.g., which are meaningful and pragmatic to assess and how often). We also recognize the challenges of including ongoing measurement of the effectiveness of EBIs; while in some cases, existing resources may provide data to monitor frequent and continued impact on health behaviors/outcomes, we realize this may not feasible across all settings.

*Example Hypothesis: Informed by a broadened, longer-term conceptualization of sustainability, the dose and nature of implementation strategies needed to initially implement an EBI will differ from the strategies needed to sustain an EBI over time (e.g. implementation strategies focused on sustainability may relate to providing proactive planning and ongoing evaluation/monitoring to manage likely changes in the implementation setting, including turnover, EHR upgrades, treatment guideline updates, changes in patient population)*.

#### 2. To Facilitate Sustainability, Planned Adaptations, and Evolutions Must Be Made Across the Life Cycle of EBIs to Respond to Changing Context

In many cases it is neither feasible nor optimal to continue to deliver the same EBI “protocol” with high fidelity, as context changes over time and across settings. There is often a need in early program stages to make planned “fidelity-consistent” adaptations that reflect diverse settings, cultures, and populations in which they are delivered (16, 18, 28). Failing to make planned cultural or contextual adaptations may have adverse impact on effectiveness, and ultimately, perpetuate health inequities (28, 29). EBIs and implementation strategies that are not aligned with and do not reflect changing community needs, culture, and context are unlikely to be sustained or have sustained impact over time.

It is also likely that there will be evolving evidence (e.g., guidelines change, new populations are exposed to EBI with varied results), setting changes (e.g., staff turnover/attrition; resources change), and shifting population health needs over time that require ongoing adaptations or refinements over time. We recommend proactively planning for adaptations, and documenting why adaptations are needed, and the extent to which EBIs and implementation strategies evolve over the life cycle of a program (16). Iterative application and measurement of RE-AIM dimensions over time enables documentation of effective adaptations to retain fit with ever evolving context. De-implementation (e.g., the removal or replacement of low-value, harmful, costly or non-evidence-based care/EBIs (21, 22), including the need to make a program and its delivery less expensive), may also be necessary and should be tracked to inform changes in implementation.

*Example Hypothesis: Settings that maintain core functions of EBIs but include proactive, planned, iterative adaptations to intervention components and implementation strategies in response to changing context and needs will be sustained longer than those that do not, and will have greater impact on reducing health inequities*.

#### 3. Assessment and Feedback on RE-AIM Indicators as an Iterative Method to Guide Adaptations

Assessment of RE-AIM dimensions can help guide settings on how to proactively monitor or adapt and may identify early indicators of sustainability challenges, including the need to “change course” to promote the sustainability of EBIs over time. Results on RE-AIM dimensions should not be assumed to be static. Thus, as explicated in Table 1, RE-AIM indicators (e.g., Reach, Effectiveness) should be measured repeatedly and iteratively when possible to provide insight into how to achieve sustained health impacts (4, 30), monitor progress, and shed light on where and when both equity and sustainability issues arise (e.g., over time, which populations and settings is the intervention reaching, and why or why not?).

These findings may impact the nature and timing of actionable solutions across RE-AIM dimensions and program life cycle —e.g., adapt the recruitment or implementation strategies. RE-AIM qualitative probes (8) can also be used to (a) help ensure that the perspectives of key stakeholders and community members are being assessed regularly; and that (b) stakeholders are being actively engaged in planning for sustainability in ways consistent with their values (e.g., “What would it take for you/your organization/your community to sustain the EBI over the long term?”) (12). Aligned with existing taxonomies of implementation strategies (e.g., evaluation/iterative strategies) (27, 31), RE-AIM can be used as a tool to complement existing quality improvement (QI) and performance management resources (e.g., PDSA cycles) (32, 33). As such, iterative application of RE-AIM can provide guidance and a conceptually-based, standardized evaluation approach to understand what is working or not; this information can be used to inform QI activities (e.g., who participates and why; where in the system is implementation of highest/lowest quality), with implications for long-term sustainability and impact.

*Example Hypothesis: Programs that iteratively assess and address RE-AIM dimensions over time to guide their sustainability planning and adaptations will have stronger sustainability outcomes (e.g. higher levels of continued delivery of EBI; higher levels of sustained behavior change across population groups) than those that do not*.

#### 4. Other Sustainability Frameworks Can Be Integrated With RE-AIM to Understand Key Sustainability Determinants

While several frameworks provide consideration of multi-level contextual factors that influence sustainability, many have been most explicitly applied in the context of implementation (34) e.g., PRISM (6, 35); and Consolidated Framework for Implementation Research [CFIR; (36)]. There may be value in also considering frameworks that have focused specifically on sustainability, including the Program Sustainability Assessment Tool [PSAT; (37)] and the Integrated Sustainability Framework [ISF; (11)], which provide a strong foundation for understanding multi-level contextual determinants of sustainability, but less guidance in measuring sustainability outcomes, or thinking explicitly about dynamic sustainability. These multi-level determinant frameworks may call attention to constructs that are particularly important to sustainability (e.g., sustainability planning, funding stability, staff retention over time), and can be integrated with RE-AIM to inform questions, measurements and actions related to contextual determinants of sustainability. For example, the ISF could be used to understand and assess multi-level aspects that may influence sustainability- e.g., “How have program champions played a role in sustaining the EBI?” Data from such qualitative assessments would preferably be integrated with quantitative measures of sustainability determinants (e.g., informed by the ISF or PSAT). It is important to recognize that sustainability determinants themselves are likely not static, and may change over time.

*Example Hypotheses: 1) Programs that explicitly address multi-level contextual determinants of sustainability will produce higher levels of sustainability and equity than those that do not; 2) Programs that address changing multi-level context and determinants of sustainability will be sustained longer than those addressing only one level*.

#### 5. Focus on Costs and Equity as Key Drivers of Sustainability Can Inform and Guide Dynamic Sustainability

Promoting health equity^1^ (39, 40) is a central part of our conceptualization and measurement of sustainability, and RE-AIM indicators should be tracked over time to identify and address inequities when they arise (further explicated in Table 1). All RE-AIM dimensions include representativeness (heterogeneity, generalization), which should be assessed across different types of patient/population subgroups of focus (e.g., by race/ethnicity, age, disability, insurance status, literacy level, social determinants of health), and settings (e.g., urban/rural, lower vs. higher resource settings). Consistent with notions of “equitable implementation” (40), it is critical to document and address inequities as they emerge across all RE-AIM dimensions. Not doing so risks maintaining or even exacerbating health inequities, and ultimately inequitable use of EBIs over time.

Issues of cost and resources required are strongly tied to health equity. For example, if an EBI is not feasible for delivery in certain settings (e.g., community health centers) due to constrained resources or insufficient staff, inequities may result. This is because these settings often reach populations that experience disproportionate social stressors and greater structural barriers to care. At the individual level, if participation requires considerable costs or burden such as travel or time off work, unintentional health inequities may result. To prevent such consequences, initial cost estimates and resource requirements should be discussed with stakeholders at the planning stage, and costs can be periodically assessed, discussed and necessary adaptations made over time (41, 42).

We consider “costs” very broadly, including understanding, planning for, and tracking economic costs, time, resources, burdens, and unintended political and social consequences (e.g., social stigma) of an EBI, especially from the perspectives of different stakeholders (e.g., implementers, administrators, community members, and patients). Recent IS research (42–44) provides suggestions for cost assessment to understand the impact on sustainability. We also encourage consideration of economic factors more broadly, including the “value” and return on investment of sustaining the EBI, and the priorities of, and value to, different stakeholders (42), including community partners.

*Example Hypotheses: 1) Programs that explicitly and repeatedly assess health equity and equitable implementation, and make iterative adjustments guided by RE-AIM will produce higher levels of sustainability than those only considering equity at the planning stage. 2) Programs that consider and monitor costs (and RE-AIM outcomes), ‘return on investment' over time, and discuss and act on these assessments in partnership with stakeholders will produce stronger sustainable outcomes than those that do not*.

### Summary

The discussion above illustrates key issues involved in extending RE-AIM to enhance sustainability. In Table 1, we outline key indicators, guiding questions, and equity and sustainability considerations in applying this extension and iterative application of RE-AIM. Consistent with complex adaptive systems (45, 46), it is more complex than the discussion makes it appear, as the various factors above and the RE-AIM dimensions are interrelated. Thus, we need to consider interactions among the issues above and across RE-AIM dimensions over time. Figure 1 highlights this complexity and considerations for cross-cutting, intersecting issues and indicators that shift (like gears) over time in different combinations to guide RE-AIM for sustainability in dynamic context across the life cycle of an EBI. This summary figure illustrates the impact of EBIs and implementation strategies on the RE-AIM dimensions, and how factors such as health equity and costs influence the likelihood of sustainability across the phases of a program.

## Discussion

This paper encourages iterative application of RE-AIM with early guidance on understanding, evaluating, and planning for sustainability, with a focus on changing context and health equity. While RE-AIM has previously been applied to promote health equity, this paper reinforces the importance of this focus within the context of sustainability. It advances the IS field beyond existing models and prior RE-AIM publications by providing: (1) consideration of planning for sustainability throughout the life cycle of an EBI and across multiple RE-AIM dimensions; (2) concrete guidance for operationalizing the dynamic and complex nature of sustainability, including the “evolvability” of an EBI and where adaptations and de-implementation may fit within this conceptualization; (3) attention to iterative measurement of RE-AIM indicators to inform and enhance sustainability, and (4) explicit consideration of health equity and costs/value as critical components of sustainability. In summary:

1. Measuring “maintenance” as a RE-AIM dimension is important, but needs to be expanded to address longer-term conceptualizations of sustainability. The conceptualization of dynamic sustainability includes consideration of “evolvability” across the life cycle of an EBI, including continued delivery of the original EBI functions and implementation strategies, adaptations, and potential de-implementation across the EBI life cycle to produce sustained and equitable health outcomes.
2. Multi-level context changes and so must EBIs and implementation strategies to meet emerging needs, resources and challenges over time. Iterative (or at least periodic) use of actionable RE-AIM assessments can guide adaptations to enhance sustainability and respond to changing context.
3. Equity (both equitable implementation across RE-AIM dimensions and health equity) and costs/value are important and understudied cross-cutting issues across all RE-AIM dimensions that impact sustainability. Researchers should assess and address these factors in planning for and facilitating long-term sustainability.

This article has both strengths and limitations. Strengths include its focus on costs and value, from the perspective of multiple stakeholders, and health equity and representativeness across all RE-AIM dimensions as key drivers of sustainability. Additionally, instead of proposing another IS model, we provide an extension of, and guidance from, a widely adopted IS framework. This paper and our recommendations address sustainability processes and planning, as well as sustainability outcomes. Finally, we make recommendations and testable hypotheses that should lead to incremental validation, revision or rejection as we refine this extension of RE-AIM. Limitations include that this proposed expansion of RE-AIM needs further empirical support. We call for future application across diverse health issues and settings, and mixed-methods research to investigate and refine this extension of RE-AIM for sustainability. There is still much to learn about sustainability, and we believe this application will provide a useful guide and addition to the IS literature.

## Author Contributions

RS and RG initially conceptualized the paper. RS took the lead in writing an initial draft. All authors contributed to reviewing, revising, and rewriting sections for this Perspective piece.

## Conflict of Interest

The authors declare that the research was conducted in the absence of any commercial or financial relationships that could be construed as a potential conflict of interest.

## Footnotes

^1^“Health equity means that everyone has a fair and just opportunity to be as healthy as possible. This requires removing obstacles to health such as poverty, discrimination, and their consequences, including powerlessness and lack of access to good jobs with fair pay, quality education and housing, safe environments, and health care…For the purposes of measurement, health equity means reducing and ultimately eliminating disparities in health and its determinants that adversely effect excluded or marginalized groups” (38).
